# Methods for verification of 3D printed anatomic model accuracy using cardiac models as an example

**DOI:** 10.1186/s41205-019-0043-1

**Published:** 2019-03-29

**Authors:** Mohammad Odeh, Dmitry Levin, Jim Inziello, Fluvio Lobo Fenoglietto, Moses Mathur, Joshua Hermsen, Jack Stubbs, Beth Ripley

**Affiliations:** 1 Institute for Simulation and Training, University of Central Florida, Orlando, FL USA; 2 Department of Medicine, Division of Cardiology, University of Washington School of Medicine, Seattle, WA USA; 3 Structural Interventional Cardiology, Virginia Mason Hospital, Edmonds, WA USA; 4 Department of Surgery, Division of Cardiothoracic Surgery, University of Wisconsin School of Medicine, Madison, WI USA; 5 VA Puget Sound Health Care System, Seattle, WA USA; 6 Department of Radiology, University of Washington School of Medicine, Seattle, WA USA

**Keywords:** Accuracy, Verification, Validation, 3D printing, Additive manufacturing, Anatomical models

## Abstract

**Background:**

Medical 3D printing has brought the manufacturing world closer to the patient’s bedside than ever before. This requires hospitals and their personnel to update their quality assurance program to more appropriately accommodate the 3D printing fabrication process and the challenges that come along with it.

**Results:**

In this paper, we explored different methods for verifying the accuracy of a 3D printed anatomical model. Methods included physical measurements, digital photographic measurements, surface scanning, photogrammetry, and computed tomography (CT) scans. The details of each verification method, as well as their benefits and challenges, are discussed.

**Conclusion:**

There are multiple methods for model verification, each with benefits and drawbacks. The choice of which method to adopt into a quality assurance program is multifactorial and will depend on the type of 3D printed models being created, the training of personnel, and what resources are available within a 3D printed laboratory.

## Introduction

3-dimensional (3D) printing is a method of fabrication that allows for the creation of graspable 3D objects from a digital blueprint. One of the most compelling use cases for 3D printing in medicine is the creation of patient-specific anatomical models for presurgical planning [1–3]. In these cases, the digital blueprint used for printing is created from a patient’s medical imaging data [4]. The resultant physical model can be given to a surgeon/ interventionalist, offering him or her an opportunity to plan the surgery before stepping into the operating room/catheterization lab. Due to positive feedback from the use of these 3D printed anatomical models during pre-surgical consultations, there has been a push to explore additional ways that 3D models can be used. Other examples of using 3D printed models in the hospital include benchtop surgical simulation [5–7], sizing of devices prior to a surgery or procedure [8–13], and designing patient-matched surgical cutting guides [14, 15] or implants [16–21].

Established quality assurance (QA) programs exist for many areas of medicine, including medical imaging. In radiology, these include Quality Control (QC) programs for ensuring optimal performance of imaging acquisition hardware [22, 23] and QA programs for dose reduction, appropriate use, radiologist interpretations, and reporting of results [24]. A handful of hospitals have led the way in adapting and extending imaging QA programs for use in 3D printing [25–27], including the creation of new phantoms that test the performance and accuracy of 3D printers and materials [1, 26]. As 3D printing programs push past enhanced visualization as a product deliverable and begin to create true medical devices, there is a need to expand the existing QA programs within hospitals to create a more robust QA program inclusive of 3D printing as a clinical resource.

Verification refers to ensuring that a part is physically made to product specifications within a given tolerance (e.g., a model of a patient’s heart matches the dimensions of the patient’s actual heart within ±1 mm), while validation ensures that the model will fulfill its intended purpose and meet the customer’s needs and expectations (e.g., the model of a patient’s heart will allow the surgeon the opportunity to practice the procedure prior to surgery). Verification that a part is built to pre-defined specifications may seem straightforward on the surface, but it can pose multiple challenges in the 3D printing space. Measuring organic shapes can be complicated; several measurement techniques have been employed to date with variable results, including manual measurements with calipers [28–30], photogrammetry, optical [31–33] and contact-based surface scanning [34], and x-ray/ CT scanning [26, 35] of a part. Another challenge is that verification requires knowing the 3D printed part’s desired dimensions, which in the case of patient-specific anatomical models means knowing the accurate dimensions of the patient’s anatomy. These reference dimensions are often impossible to physically measure if the anatomy is internal, and therefore the reference standard for a given 3D model or part is often based on medical imaging, which becomes a stand-in for ground truth.

In this paper, we explore several measurement methods for verifying the accuracy of 3D-printed cardiac models and discuss some of the unique challenges we encountered with each method. This work is not meant to determine the superiority of one technique over the other nor to make a statement about what technique should ultimately be used. Instead, it is meant as an overview and a conversation starter concerning the challenges inherent in part verification.

## Methods

### Subjects

Patient-specific models were created at the University of Washington School of Medicine as part of an ongoing, IRB-approved research project to determine the utility of 3D printed heart models for pre-procedural planning. A subset of these hearts underwent additional quality assurance testing, along with 3D printed models of cadaveric donor hearts, to establish a standard operating procedure for model verification.

### Creation of 3D printed anatomical models from patient CT scans

Cardiac CT scans were acquired using a GE Revolution scanner (GE, Waukesha, WI) in axial plane at 0.625 mm slice thickness, 100–120 kV, and variable mA with dose modulation. Pixel spacing was 0.488 × 0.488 mm, with image matrix 512 × 512. Images were reconstructed using the GE “Standard” convolution kernel.

Next, the Digital Imaging and Communications in Medicine (DICOM) images were imported into Mimics Medical software version 21.0 (Materialise, Leuven Belgium) and were manually segmented using thresholding-based strategies. For blood pool segmentation, the blood pool mask was edited to exclude non-cardiac structures (e.g. ribs, pulmonary vasculature). For myocardium models, a second mask was segmented based on myocardial Hounsfield units and the blood pool mask was subtracted from the myocardial mask. In a subset of cases included in this study, the CT scanner table was also segmented using a thresholding strategy. Heart volume and CT table masks were exported from Mimics Medical into 3-Matic Medical Software (Version 13.0) as STL files for further STL processing, including smoothing and correction of vertex errors. Once STL editing was complete, all files were imported back into Mimics software for final qualitative verification of positioning and mask accuracy with respect to the original DICOM imaging.

Finally, all models were 3D-printed on a Form2 printer (Formlabs, Cambridge, MA). Models were printed out of clear, white and gray resins.

### Creation of 3D printed anatomical models from cadaveric hearts

Cadaveric hearts were placed into plastic containers and fully submerged in a saline bath spiked with iodinated contrast. The contrast: saline ratio was titrated so that the saline bath measured approximately 400 Hounsfield units (±25 HU, Standard Deviation) on test scans. This provided sufficient contrast between the cadaveric heart tissue and the surrounding saline for segmentation. Cadaveric hearts were imaged using a GE Revolution scanner (GE, Waukesha, WI) in the axial plane at 0.625 mm slice thickness, 100 kV, and 375 mA. Pixel spacing was 0.488 × 0.488 mm, with image matrix 512 × 512. Images were reconstructed using the GE “Standard” convolution kernel.

Segmentation methods and STL file creation were essentially as described above. Slicing software used for g-code creation was Cura (Ultimaking Ltd., London, England). 3D printed heart models were printed on a material extrusion printer (Printrbot simple; Printrbot, Sacramento CA) using flexible white filament (Ninjaflex; Ninjatek, Manheim, PA).

### Measurement of discrete features on 3D printed models

**Fig. 1:**
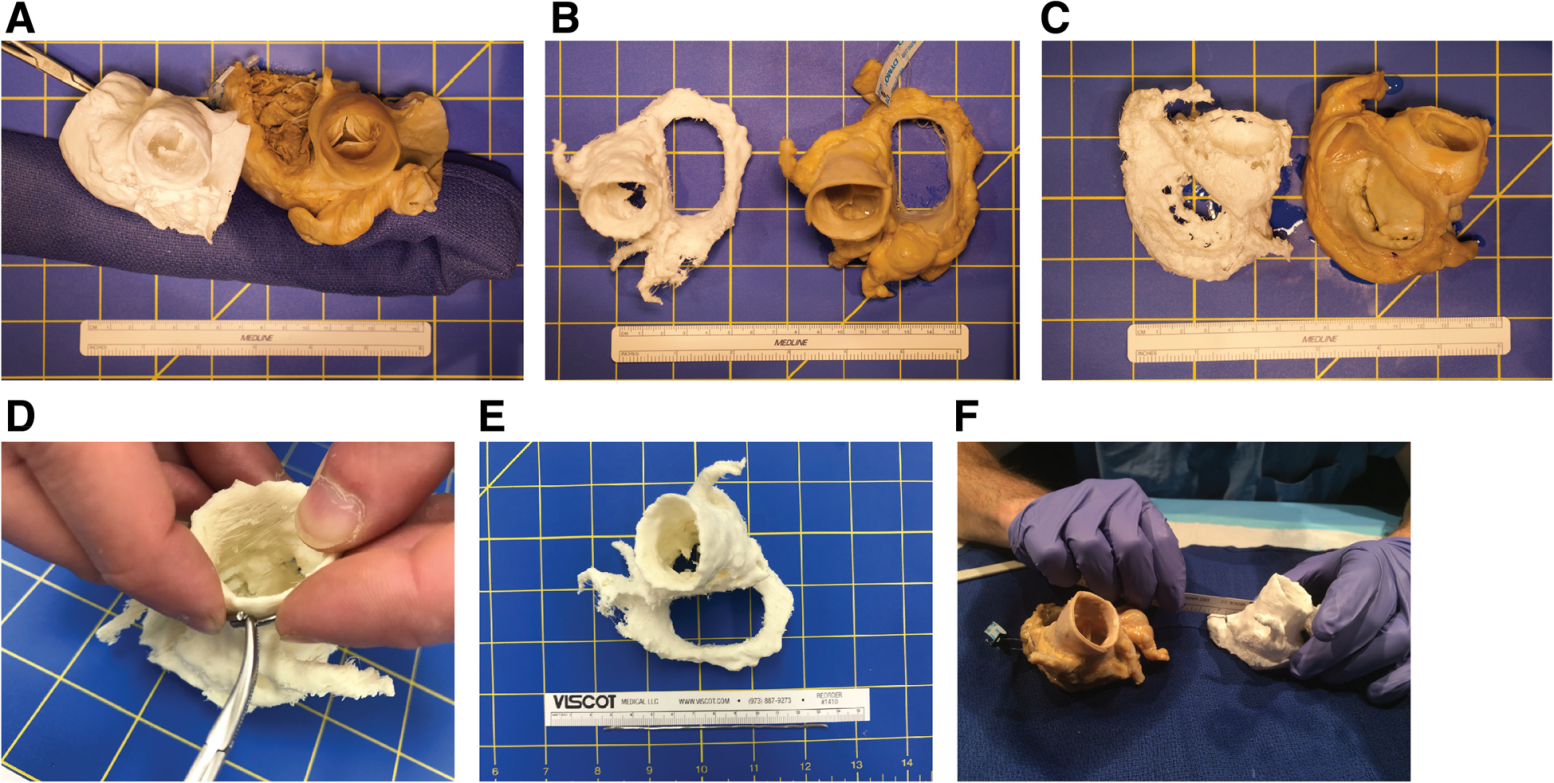
Physical measurements of 3D printed cardiac models and cadaveric hearts. **a-c** Three examples of cadaveric hearts and their corresponding 3D printed models, used for measuring features of the aortic valve (**a**, **b**) and the mitral valve (**b**, **c**). **d-e** Measurement of perimeters was accomplished using a malleable metal wire which was marked with a needle driver and then straightened and measured against a ruler. **f** Organic shapes present challenges to measurement with linear rulers or calipers

In the first set of experiments, we explored the feasibility of physically measuring complex organic shapes. Discrete features of interest were measured on 5 cadaveric hearts and their corresponding 3D printed models. Features were as follows: sinotubular junction perimeter (3 models), aortic annulus maximum and minimum diameter (3 models), mitral valve maximum and minimum diameter (2 models), and mitral valve annulus perimeter (2 models). Diameter measurements were made with sliding digital calipers (Neiko, Taiwan) and recorded to the nearest tenth of a millimeter; perimeter measurements were made with a bendable wire marked at length with a needle driver and subsequently measured against a ruler to the nearest tenth of a millimeter (Fig. 1). Four experimenters measured each feature (cardiothoracic surgeon JH, interventional cardiologist MM, radiologist with cardiovascular imaging training BR, and cardiac anatomist DL).

**Fig. 2:**
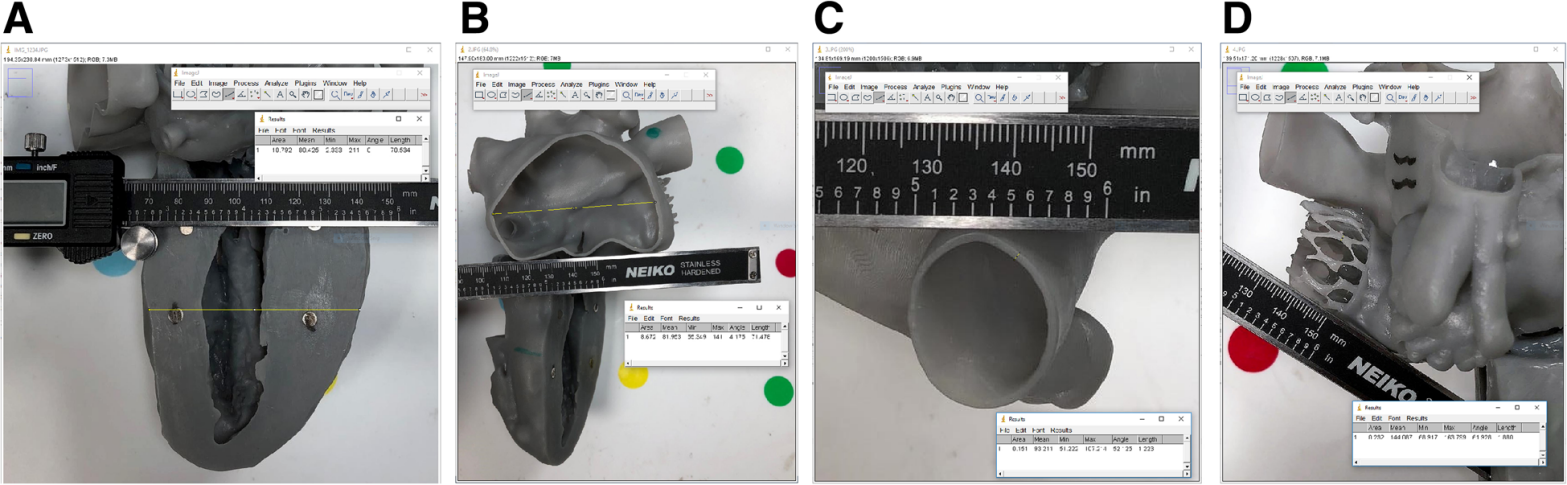
Digital photographic measurement technique. **a-d** Four discrete 3D model features (**a** Feature 1, **b** Feature 2, **c** Feature 3, and **d** Feature 4) were chosen for measurement, based on their accessibility on an exterior surface and the fact that they had distinguishing features that would allow for repeat measurements. All digital photographs were calibrated before measurement. This was achieved by placing a marker of known length at the same height and angle as the feature of interest before photographing the model. Once the photograph was imported into an image analysis software (in this case, ImageJ), the length of the known marker was measured in pixels, and a conversion factor was calculated for pixels to millimeters. Of note, insufficient illumination for feature 3 (panel **c**) caused blending of edges, most pronounced along the inferior aspect of the photograph. This affected accuracy of measurements for this feature (see Table 2)

In the second set of experiments, we explored two different techniques for measuring discrete features on 3D models - manual measurements made with a digital caliper and measurements made on digital photographs. We identified four discrete features of interest that were accessible by calipers (Fig. 2) and obtained a total of 7 linear measurements from these 4 features (wall thickness, maximum and minimum diameter of openings in the model). Manual and digital photographic measurements were made by a single experimenter (M.O.) blinded to actual feature dimensions. Digital photographs were acquired by placing a marker of known length parallel to and at the same height as the feature in the model and then capturing a digital photograph using an iPhone X with the stock camera application (12Megapixels and f/1.8 aperture). The photographs were imported into Image J digital measurement software (National Institutes of Health, Bethesda, Maryland) and were calibrated by performing a ratio between the pixel count and known physical length of the marker. The linear measurement tool in image J was then used to repeat the same 7 measurements that were made with calipers.

### Measurement of discrete features from an STL file

**Fig. 3:**
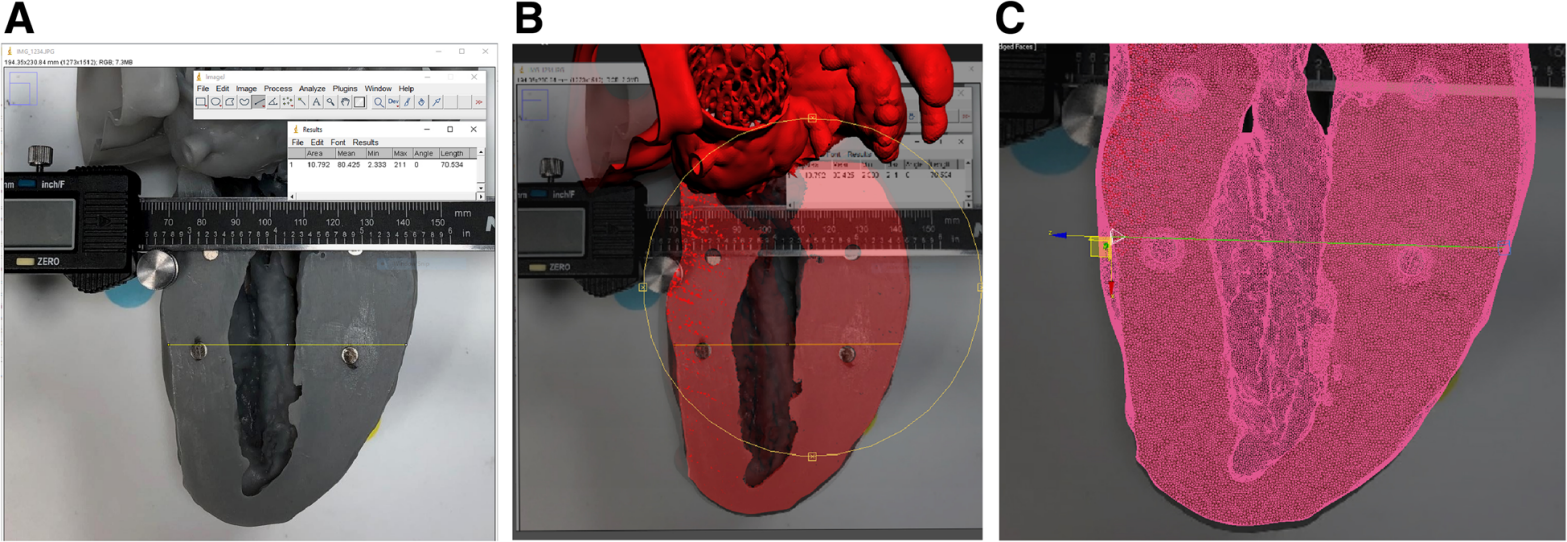
Vertex to vertex edge STL file measurement. **a** Digital photograph exported from Image J with measurement included for landmark reference. **b** The digital photograph was imported into Autodesk 3D Studio Max and overlaid on the STL file (rendered in red). The STL file was carefully aligned so that the meshed feature of interest aligned with the photograph. **c** Vertex to vertex edge measurement was made of the meshed STL file

We investigated the feasibility of making discrete measurements on an STL file to serve as the standard against which to compare physical and digital photographic measurements of 3D printed models. Measurements of discrete features on the original model STL file were made in Autodesk 3D Studio Max 2018 (Autodesk, San Rafael, CA), a professional 3D computer graphics program for digital vertex to vertex measurement. Digital photographs with embedded measurements were exported from Image J, imported into Autodesk 3D Studio Max, and overlaid on the STL file for reference to ensure that the STL file features were measured at the same location as the digital photographic measurements. A sample of the output produced by 3D Studio Max along with the steps is presented in Fig. 3.

### Comparison of discrete feature measurements across measurements styles

We did not design or power this study to formally compare differences between the measurement techniques described above, and therefore no formal statistical analysis was performed. Instead, the data is presented in raw table format and expressed as standard deviation or percent error where applicable. For digital caliper and digital photographic measurements compared against STL file measurements, the percent error was defined as the absolute value of (STL measurement – experimental measurement) divided by STL measurement and multiplied by 100.

### Photogrammetry and surface scanning of 3D printed models

**Fig. 4:**
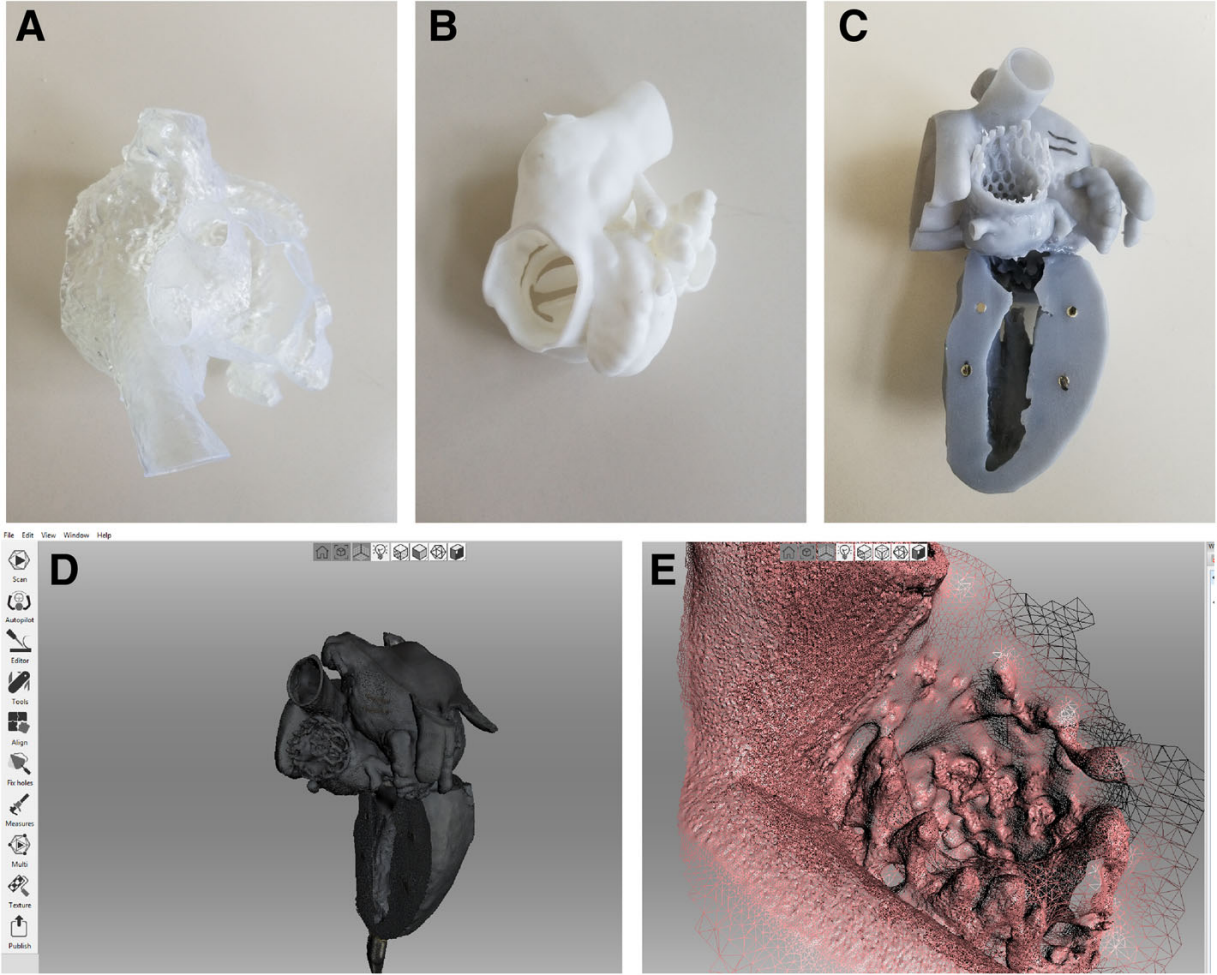
Surface scanning success was dependent on model surface characteristics. Models printed in clear (**a**), white (**b**) and gray (**c**) resin were used for surface scan testing. Clear and white models were not successfully scanned, due to challenges related to scanning of transparent (clear model) and reflective (white model) surfaces. The model printed in the gray resin was successfully scanned, with the resultant surface model shown in (**d**). However, fine model features were degraded in the surface scan, such as the transcatheter aortic valve depicted in (**e**)

We explored two methods for optical imaging of 3D model surfaces - photogrammetry and surface scanning. We chose a total of 3 models (printed in gray, white and clear resin, respectively) for optical imaging experiments (Fig. 4). For photogrammetry, a series of photographs were taken at different angles and orientations (a total of 40 on average) with an iPhone X with the stock camera application (12MP and f/1.8 aperture). The auto white-balance/focus was kept on to maintain consistency between all images taken. Images were taken at a constant distance from the object of interest. Images were processed using VisualSFM (a GUI application for 3D reconstruction created by Changchang Wu, available at http://ccwu.me/vsfm/) to create a computed set of three-dimensional coordinates needed for photogrammetry. For surface scanning, the same 3 models were scanned using a structured light Artec Space Spider scanner with a resolution up to 0.1 mm (Artec, Santa Clara, CA).

### CT scanning of 3D printed models

**Fig. 5:**
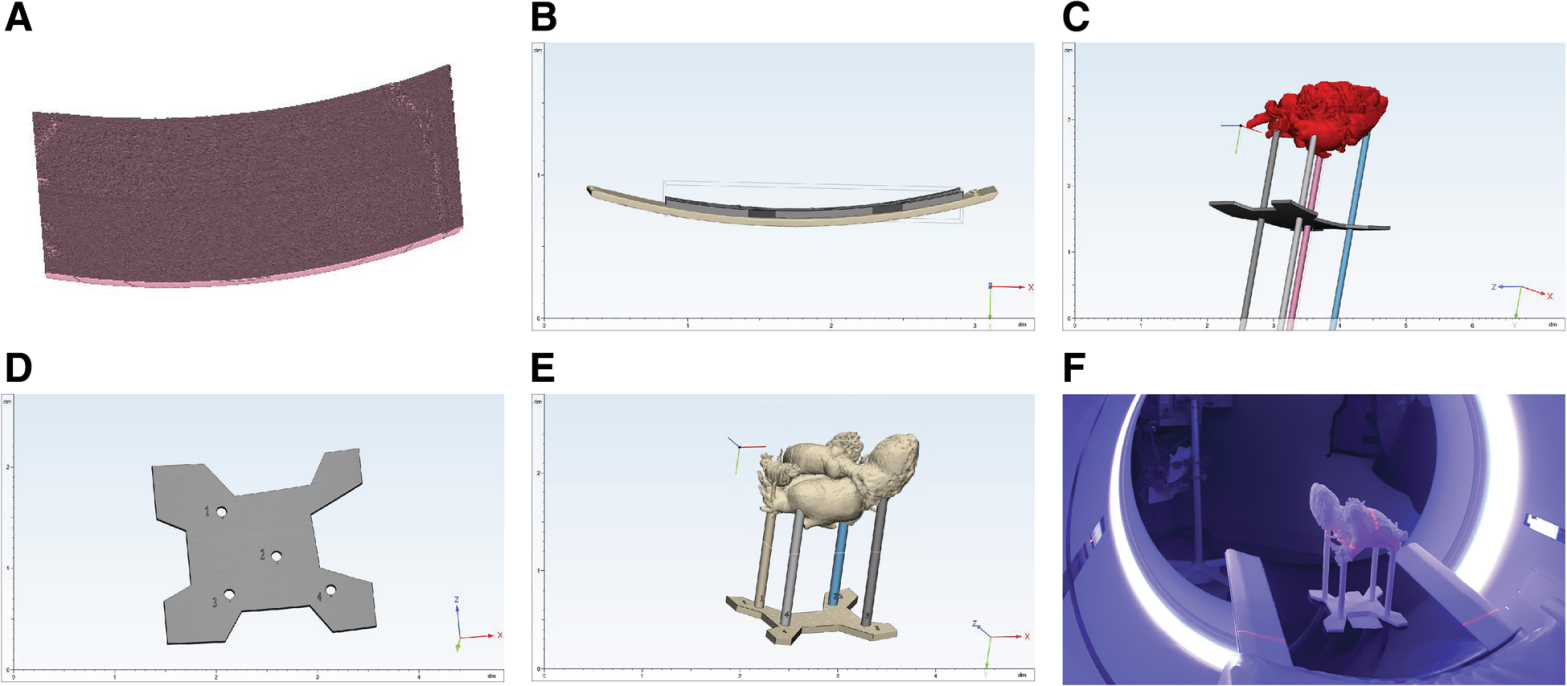
3D model-specific heart holder for optimal positioning during CT scanning. **a** The CT table was segmented and exported as an STL file at the time of model segmentation and creation. **b** A custom table adaptor piece created in CAD software was designed to sit flush with the CT Table. **c** The corresponding heart model was imported into CAD software, and 4 cylinders were created and positioned so that they intersected the model and the table adaptor piece. **d** Holes fitted to the cylinders with 0.1 mm offset were created in the model, and the table adaptor piece and the cylinders were shortened to the appropriate length. **e** Example of finished model-specific heart holder in CAD software. **f** 3D printed heart and heart holder positioned in the CT gantry in the same orientation that the patient was scanned in (feet first)

3D printed heart models were CT scanned and compared to patient DICOM datasets. A unique heart holder was created for each model to ensure that the model was scanned in the same spatial orientation within the CT scanner as the patient’s heart in the original scan. The first step in creating the heart holder was segmenting the CT table from the patient DICOM at the time of heart segmentation. Segmented CT tables were imported into 3-Matic as STL files, as described above. The table was cloned and offset in the Y-axis by the table thickness (measured on the DICOM images using standard picture archiving and communication system [PACS] measurement tools) so that the cloned table sat perfectly flush with the CT table. Next, a set of cylinders were created which transected both the heart and the cloned table. The cylinders were labeled 1–4 and were notched to ensure appropriate orientation with the table piece. Holes with 0.1 mm offset were created by Boolean subtracting the cylinders from the heart model as well as the table piece (Fig. 5). One heart model and accompanying heart holder was printed on both a Form2 printer as well as a Connex3 Objet 350 printer (Stratasys, Rehovot, Israel) to allow for comparison of the technique across printers.

Heart models (in their respective heart holders) were scanned on the same Revolution Scanner (GE, Waukesha WI) that the initial CT images were acquired on with the following parameters: axial mode with 0.625 mm slice thickness, at 120 kVP, 375 mA, reconstructed using GE “Standard” convolution kernel. Pixel spacing was 0.488 × 0.488 mm with image matrix 512 × 512.

### Alignment of patient DICOM and 3D model DICOM datasets

We aligned the patient DICOM dataset and the 3D model DICOM dataset in Osirix MD (Pixmeo, Geneva, Switzerland) by creating a fusion image of the two DICOM files. Detailed instructions for making fusion images are available online (https://en.wikibooks.org/wiki/Online_OsiriX_Documentation/Making_fusion_images).

The heart holder positioned the model in the same spatial coordinates and alignment as the patient’s heart on the initial scan, and thus there was no requirement for non-rigid deformation. Minimal differences in X, Y and Z coordinates between the two DICOM datasets were corrected using the pan and rotate tools in Osirix.

## Results

### Physical measurements of discrete 3D printed model features

Physical measurements of discrete model features presented some challenges (Fig. 1). Nonlinear measurements such as perimeters could not be accomplished with straight rulers or digital calipers. Instead, we used a thin malleable wire to mark the perimeter length and then straightened and measured the wire. The standard deviation (SD) between observers for perimeter measurements was approximately twice as large as the SD for linear measurements made with calipers (SD range of ±2.1 mm to ±9.2 mm for perimeter measurements, compared to SD range of ±0.8 mm to ±4.4 mm for linear measurements with calipers) (Table 1). Physical measurements were not possible for features that were internal to the model or recessed beyond the reach of calipers (e.g., the aortic annulus in a model that includes portions of the ascending aorta and the left ventricular outflow tract).

**Table 1:** Physical measurements of cadaveric hearts and 3D printed models

Cardiac Valve	Feature	Model #	3D model measurement/ cadaveric model measurement in mm (% difference)				± Standard Deviation across 4 observers		
			Observer 1	Observer 2	Observer 3	Observer 4	3D Model (mm)	Cadaveric heart (mm)	% Difference
Aortic	STJ perimeter	1	105.0/104.0 (101.0)	106.0/103.0 (102.9)	106.0/101.0 (105.0)	97.0/106.0 (92.0)	4.4	2.1	5.7
Aortic	STJ perimeter	2	103.0/98.0 (105.1)	105.0/100.0 (105)	87.0/89.0 (97.8)	107.0/97.0 (110.3)	9.2	4.8	5.1
Aortic	STJ perimeter	3	101.0/106.0 (95.3)	103.0/102.0 (101.0)	100.0/103.0 (97.1)	106.0/111.0 (95.5)	2.6	4.0	2.6
Aortic	Max Diameter	1	34.4/32.8 (104.9)	34.6/34.5 (100.3)	35.1/34.1 (102.9)	32.1/33.2 (96.7)	1.3	0.8	3.6
Aortic	Max Diameter	2	33.3/32.5 (102.5)	34.5/32.4 (106.5)	29.6/28.1 (105.3)	34.4/34.4 (100.0)	2.3	2.7	2.9
Aortic	Max Diameter	3	32.5/31.1 (104.5)	32.3/31.0 (104.2)	29.9/32.7 (91.4)	35.3/35.6 (99.0)	2.2	2.2	6.1
Aortic	Min diameter	1	29.6/29.7 (99.6)	30.6/29.9 (102.3)	29.4/31.0 (94.8)	27.3/29.2 (93.7)	1.4	0.8	4.1
Aortic	Min diameter	2	27.6/25.6 (107.8)	27.6/26.4 (104.5)	23.9/23.4 (102.1)	27.4/27.4 (100.0)	1.8	1.7	3.4
Aortic	Min diameter	3	28.0/27.8 (100.7)	28.1/28.4 (98.9)	31.8/26.6 (119.5)	29.6/30.2 (98.1)	1.8	1.5	10.2
Mitral	Annulus perimeter	4	120.0/125.0 (96.0)	116.0/127.0 (91.3)	121.0/130.0 (93.1)	124.0/126.0 (98.4)	3.3	2.2	3.1
Mitral	Annulus perimeter	5	122.0/125.0 (97.6)	112.0/119.0 (94.1)	110.0/110.0 (100.0)	101.0/121.0 (83.5)	8.6	6.3	7.3
Mitral	Max Diameter	4	43.5/45.2 (96.2)	43.5/43.2 (100.7)	43.3/45.3 (95.6)	37.9/37.9 (100.0)	2.8	3.5	2.6
Mitral	Max Diameter	5	40.2/43.6 (92.2)	40.0/40.9 (97.8)	47.5/45.3 (104.9)	40.4/35.3 (114.7)	3.6	4.4	9.7
Mitral	Min diameter	4	26.5/28.3 (93.6)	25.1/24.9 (100.8)	25.3/27.5 (92.0)	27.3/27.3 (100)	1.0	1.5	4.4
Mitral	Min diameter	5	23.9/24.5 (97.6)	24.0/28.3 (84.8)	25.6/30.3 (84.5)	23.0/29.5 (77.9)	1.1	2.6	8.2

Measurements were made of aortic valves on 3 hearts and mitral valves on 2 hearts by 4 observers. Aortic valve features measured included the perimeter of the sinotubular junction (STJ), which is important in pre-procedural planning for transcatheter aortic valve replacement (TAVR), and the maximum and minimum valve diameter. Mitral valve features included the perimeter of the mitral annulus, as well as the maximum and minimum diameter of the mitral valve. 3D model and cadaveric heart measurements are presented in millimeters (mm), along with the percentage difference between the 3D model and accompanying cadaveric heart measurements, for each observer. Percentage differences exceeding 100% indicate that the model measurement was larger than the cadaveric heart measurement. The standard deviation for measurements across the 4 observers is listed in the right-hand columns for 3D model feature measurement, cadaveric heart measurement, and % difference between the 3D model and cadaveric heart measurements

### Digital photography-based measurements of discrete 3D printed model features

Digital photography-based measurements could only be made for external features and were constrained by the requirement that a measurement marker be positioned in the same spatial plane as the feature of interest. We found that appropriate lighting was critical for measurement accuracy. For example, improper illumination in the digital photograph of feature 3 led to blending of edges and inaccurate measurements for this feature when compared to the reference STL (Fig. 2c, Table 2).

**Table 2:** Measurements of discrete 3D model features

		Length (mm)			% Error	
Feature	Geometry	Caliper	Digital	STL measure	Caliper	Digital
1	Maximum Diameter	69.64	70.53	69.98	0.48	0.79
2	Wall Thickness	1.78	1.69	1.70	4.46	1.12
	Maximum Diameter	70.90	71.48	70.83	0.10	0.92
	Minimum Diameter	50.82	52.30	51.12	0.58	2.31
3	Wall Thickness	1.44	1.22	1.40	2.86	12.64
	Maximum Diameter	23.56	20.72	23.09	2.04	10.26
4	Wall Thickness	1.94	1.88	2.06	5.64	8.56

A total of 4 different features were measured on a 3D printed model using physical (calipers) and digital photographic measurement strategies. The features were additionally measured from the source model STL file using Autodesk 3D Studio Max. A total of 7 different geometries (model wall thickness and model opening diameters) were measured

### Surface scanning and photogrammetry of 3D printed models

3D printed model surfaces were variably challenging to capture with structured light surface scanning and photogrammetry approaches. The clear resin model was not amenable to surface scanning because the emitted light from the 3D scanner passed through the model and was not reflected onto the scanner’s registration sensor; therefore, no points were registered on the surface of the object. The white resin model had a reflective surface which caused light to scatter and resulted in a sparse and inaccurate point cloud. Surface scanning of the gray resin model was largely successful, but some fine features were blurred (Fig. 4). For photogrammetry approaches, the calculated point cloud was dependent on the number of images obtained, the percentage of overlap between images, appropriate centering of the object of interest in the image frame, and the consistency in lighting (no hard shadows). As with surface scanning, almost no surface points were collected from the model printed in clear resin. For the white resin and gray resin models, the point cloud was sparse, with more useable data obtained from the gray model relative to the white. In the end, however, the point cloud was not sufficient for comparison to the model STL files.

### CT scanning of 3D printed models

**Fig. 6:**
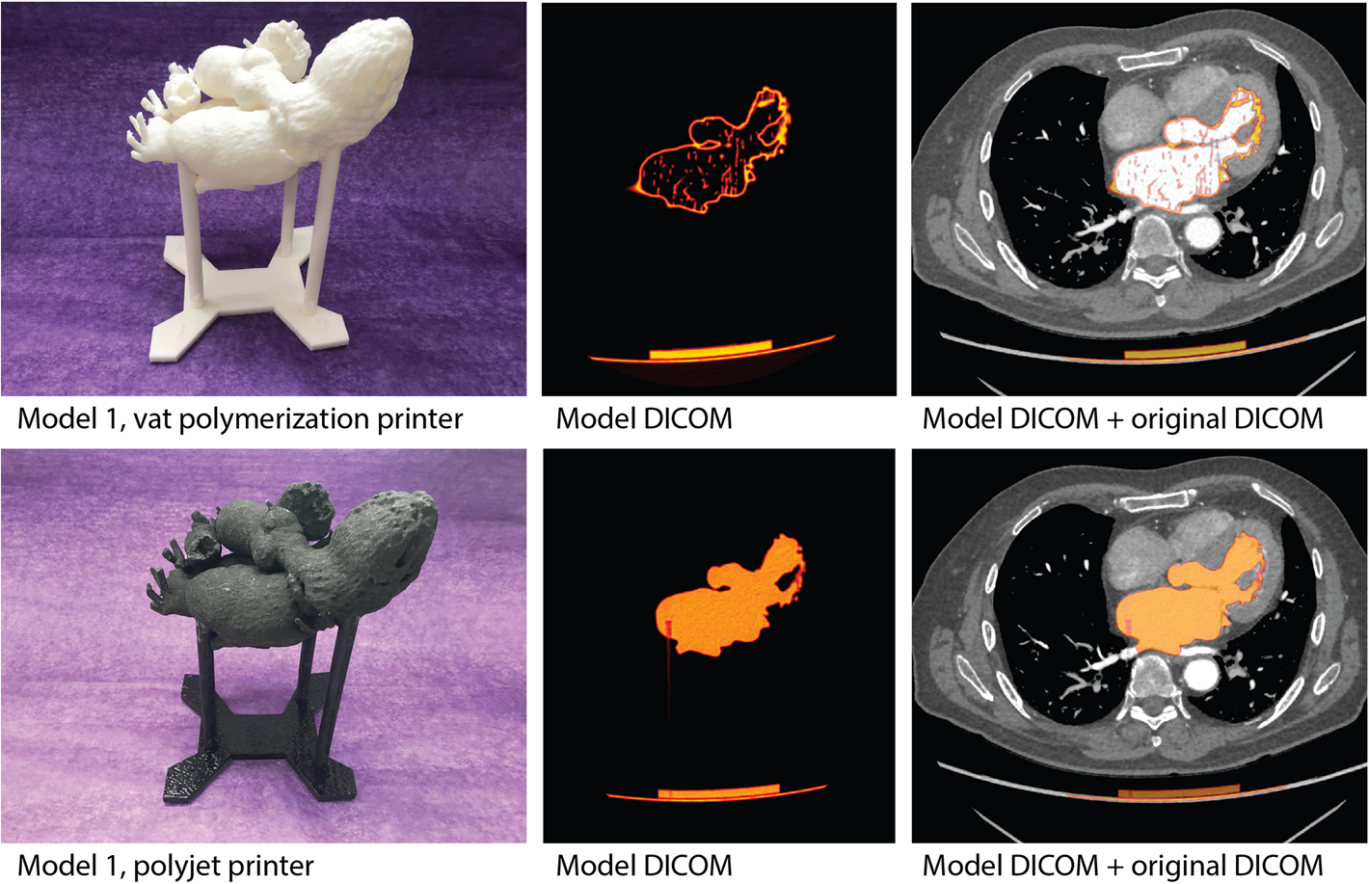
3D heart models CT scanned using a custom heart holder. Heart models were CT scanned for an end-to-end verification of the final 3D model back to the original DICOM images. A custom heart holder was created from source DICOM images to ensure that the model was imaged in the same orientation and spatial position as the original anatomy. Top row: (left) 3D printed heart and 3D printed holder, created on a Form2 printer, (middle) DICOM image from the CT scan of the 3D model, and (right) DICOM image from the original patient study, with the 3D model DICOM overlaid for comparison. (Of note, this model was hollowed to save material, and the specular foci within the center of the model represent internal support structures.) Bottom row: same as above, but in this case, the heart and holder were printed on a Connex 3 Objet 350 printer. This strategy allowed for verification of the model creation pipeline for 2 separate printing techniques. (Of note, this model is filled with internal support material, which on this printer is laid down uniformly, in contrast to the strut-like supports of the Form2 model)

**Fig. 7:**
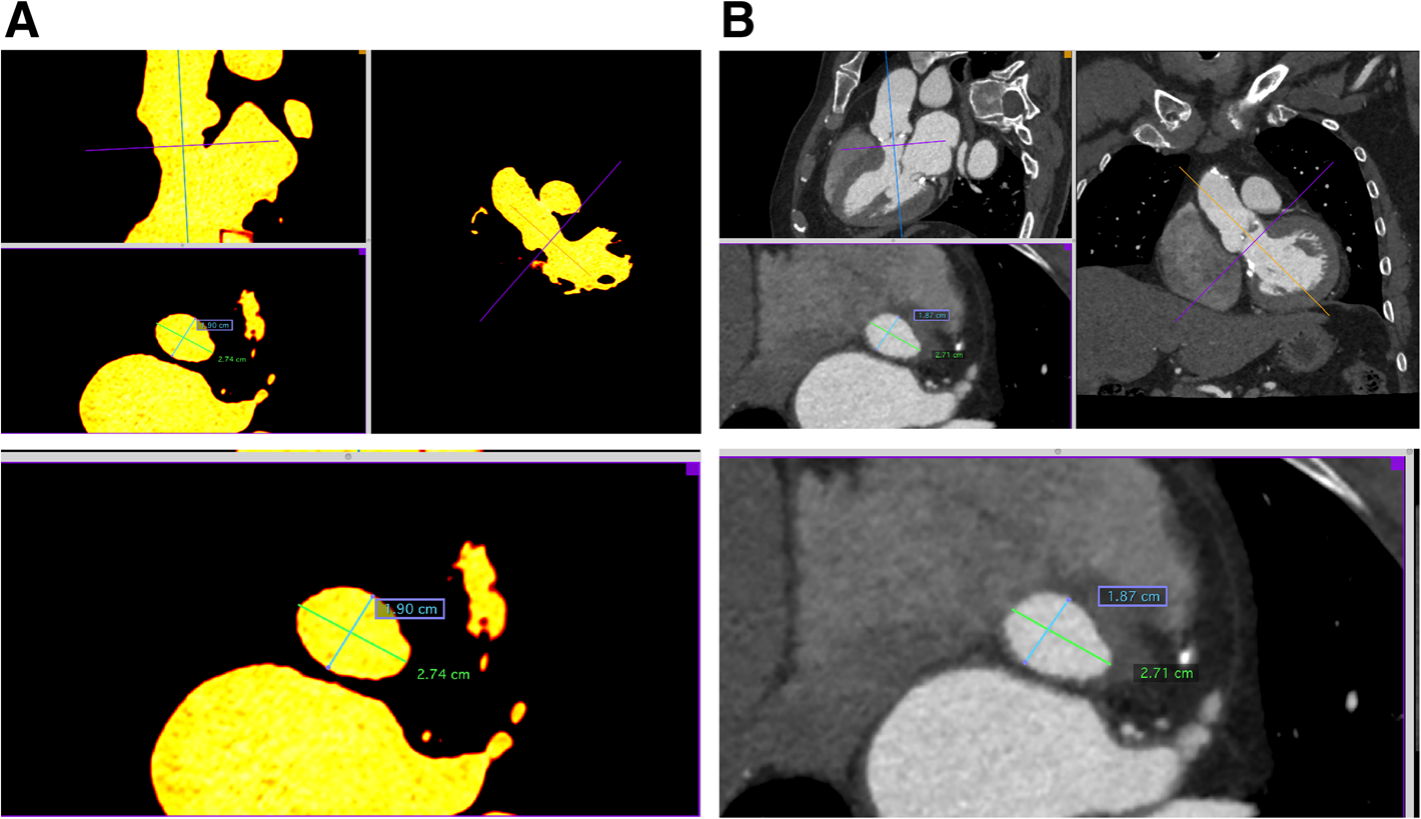
Quantitative measurements of a 3D printed model by CT. Measurements of critical features (e.g., left ventricular outflow tract just inferior to aortic annulus) were made on final 3D printed model DICOM images (**a**, in yellow) and checked against the source patient DICOM data (**b**)

CT scanning of 3D models was relatively simple to perform but required the creation of a model-specific holder to minimize image registration challenges. With the heart holder, DICOM image stacks of the 3D model and the source DICOM images could be aligned using simple pan and rotate tools in Osirix MD. A qualitative check of the model contours relative to the anatomy of interest was accomplished by scrolling through the dataset (Fig. 6). Quantitative measurements were also made on the final 3D model DICOM images for critical features (Fig. 7).

## Discussion

A quality assurance (QA) program takes years to establish and build and is comprised of numerous components, each with its inherent intricacies. Here, we explored one component of a QA program - verification of part accuracy. Verification requires measurement of final model dimensions and comparison of those measurements to the physical geometry of the patient’s anatomy. In cases of internal anatomy, the patient’s medical imaging often serves as a proxy for the actual anatomy. The substitution of imaging data for ground truth inherently introduces some degree of error into the verification process, which is mitigated by established radiology department QC/QA practices for ensuring image accuracy. It is important to remember that the quality of the imaging always constrains the quality of the model.

**Fig. 8:**
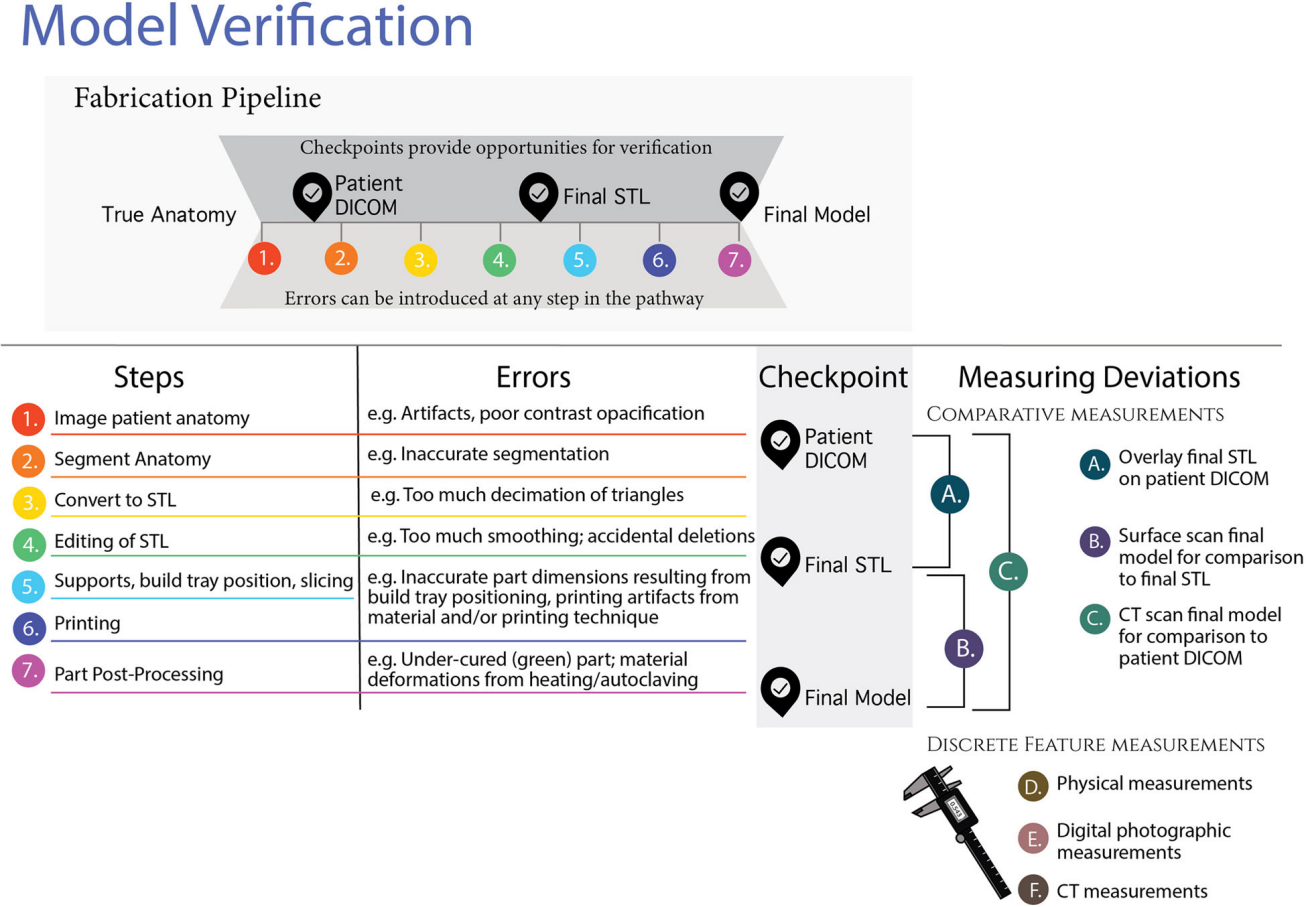
Infographic overview of 3D printed model / part verification

Unfortunately, each step in the process of 3D-printing an anatomical model can introduce error (Fig. 8). Because of this, it is necessary to minimize and continue to monitor errors created by the fabrication process to ensure safe and impactful use of these models in a clinical setting. Checkpoints in the design process are often used for intermediate verification checks. For example, overlaying a final STL on the original DICOM image or comparing a point cloud obtained from surface scanning of the final model to the STL file are examples of these types of checks. The challenge is that none of these methods fully verify the model, since they depend on derivatives of the patient’s anatomy and are, in of themselves, subject to the introduction of errors.

There are multiple strategies for part measurement, including manual measurements, digital photographic measurements, surface imaging strategies (3D surface scanning and photogrammetry) and CT scanning. We sampled various options as part of feasibility testing in building our quality assurance program. It is important to emphasize that the data presented here is not a comprehensive assessment of the accuracy of these measurements in of themselves (which has been done elsewhere). Instead, the goal was to discover the strengths and weaknesses of each technology in our hands to find the best fit for clinical heart model verification in our hospital-based program (Table 3).

**Table 3:** Different measurement techniques have advantages and disadvantages

Technique	Overview	Advantages	Disadvantage
Manual measurements	Measuring instruments such as digital calipers are used to physically measure features.	• Cost effective • Straightforward technique	• Can be difficult to access internal structures • Overtightening of calipers can deform flexible models • Non-linear measurements such as perimeters cannot be accomplished with calipers
Digital photographic measurements	The process requires photographing a feature of interest with a reference scale in the same plane. The scale consists of an object of known size which can be used to determine the pixel/dimension ratio. The ratio is then used to measure the feature of interest.	• Simple set-up • Cost effective • Nonlinear measurements are possible, such as perimeter measurements	• Differences in illumination can introduce error • Reference feature and/or scale must be on the same plane as the feature of interest (or the difference must be accounted for)
3D Scanning	This method uses lasers or structured light to collect information about an object’s surface. This information is assembled as a point cloud (digital 3D coordinates) which can be translated into a digital 3-dimensional surface model of the structure.	• High resolution • portable	• Scanners can be expensive: Optical pattern projection systems range from $1000- $100,000. Laser scanning systems range from $25,000 - $1,000,000. • There is a learning curve for proper scanning technique • Clear and reflective model surfaces can be challenging to scan
Photogrammetry	This method uses digital images of a 3D object, taken at different angles, to generate a 3D reconstruction using image registration. The method has become very popular in the recent years thanks to the widespread implementation of high-resolution cameras in mobile devices.	• Cost effective	• Requires understanding of image processing principles • Requires familiarity with an array of software packages (e.g., Visual SFM, Meshlab)
CT scanning	Computed tomography uses x-rays to produce 2-dimensional images that are stacked to produce a 3D volume.	• Can measure geometry of internal structures • Fast and accessible in most hospitals	• Expensive • Requires image registration to original DICOM

Physical measurements presented early challenges, as critical non-linear cardiac anatomy such as the aortic and mitral valve annulus could not be measured with calipers or a ruler. We used a malleable wire to mark perimeter lengths but found the inter-observer variability to be more than twice that of measurements made with calipers. A portion of that variability is likely technical, as small bends in the wire that were not perfectly smoothed out introduced error. More importantly, however, it required personnel with extensive anatomical training to make the measurements, and concepts such as how to measure the mitral valve annulus were dependent on the background and training of the person performing the measurements. Digital photographic measurements could potentially mitigate the challenges of nonlinear measurements but were still limited to being utilized for external structures, and the requirements for marker placement and camera set-up were non-trivial.

We investigated two methods for measuring the surface of models- structured light surface scanning and photogrammetry. We learned that model material color heavily influenced the success of these methods in our hands; we were unable to obtain usable datasets for clear and white resin models. Marking the models with talcum powder or a developer spray may have helped with scanning, but we were concerned that those options would have affected the overall part geometry, and for that reason, they were not used in this study. While there were multiple additional steps we could have taken to improve results with these techniques, including testing different types of scanners (for surface scanning) or improving image acquisition techniques (for photogrammetry), we determined that the time and staff training needed was outside of the scope of what was feasible for our program. These techniques are likely to perform well in hospital-based programs that have optimized the process and have staff who are experienced with these techniques.

We found that CT scanning circumvented many of the challenges faced by other measurement techniques. Perhaps most importantly, CT scanning of the 3D printed model allowed end-to-end verification of the entire 3D printing workflow by comparing the final 3D printed model directly to the patient’s anatomy in the original DICOM images (Fig. 8). Overlaid 3D printed model and patient DICOM images were easily scrolled through by a radiologist, allowing for overall qualitative confirmation of accuracy and additional quantitative measurements of critical parts of the model at the discretion of the radiologist (e.g., aortic annulus measurements in a model intended for pre-procedural planning for transcatheter aortic valve replacement, Fig. 7). An additional benefit was that our radiology staff have baseline expertise in CT technology, and therefore required no additional training to incorporate model verification into their daily workflow.

One potential challenge of using CT to verify 3D models is the requirement for DICOM image registration. Fiducial markers or distinct landmarks are often necessary for image registration, but these require foresight to add to the patient before a scan. To minimize the challenges of landmark registration, we used the CT table itself as a fiducial marker. This strategy allowed for rigid image registration with a minimum of time and effort (less than 5 min, on average), but at the cost of the design and printing time that came with the creation of the heart holder. A portion of the design work may be automated going forward.

### Limitations

This work is limited by the small sample size and lack of statistical analysis. We did not test contact-dependent methods of surface analysis, such as a coordinate measurement machine. We did not troubleshoot initial challenges with surface scanning and photogrammetry approaches, and we do not present the steps required for registration of surface point cloud data with STL files needed for model verification with these strategies. The model-specific heart holder presented here for CT scanning is a prototype, and has not been tested with flexible models or anatomy outside of cardiac models.. Finally, while the methods described are detailed with respect to cardiac models, it is very likely that modifications to ensure accuracy would be necessary for other clinically appropriate 3D printed models. [36]

## Conclusion

Verification of 3D printed model accuracy is an essential, but nontrivial, component of a quality assurance program. Numerous methods for measuring 3D printed model dimensions exist, each with benefits and drawbacks. The choice of verification method should be made after considering the unique clinical requirements of the 3D model being verified, as well as the expertise and preferences of program staff. Specific recommendations to the reader on the number or frequency of models to verify for a given workflow is beyond our purview; instead, readers should look to existing regulatory guidance [37, 38].
